# Docking Studies, Synthesis, and *In-vitro* Evaluation of Novel Oximes Based on Nitrones as Reactivators of Inhibited Acetylcholinesterase

**Published:** 2017

**Authors:** Seyed Ayoub Hosseini, Abolghasem Moghimi, Maryam Iman, Firoz Ebrahimi

**Affiliations:** a *Department of Chemistry, Faculty of Science, Imam Hossein University, Tehran, Iran. *; b *Chemical Injuries Research Center, Baqiyatallah University of Medical Sciences, Tehran, Iran.*; c *Department of Pharmaceutics, Faculty of pharmacy, Baqiyatallah University of Medical Sciences, Tehran, Iran. *; d *Department of Biology, Faculty of Science, Imam Hossein University, Tehran, Iran.*

**Keywords:** Reactivators, Oximes, Molecular docking, Nitrones, Organophosphorus compounds

## Abstract

Acetylcholinesterase has important role in synaptic cleft. It breaks down the acetylcholineat cholinergic synapsesand terminates the cholinergic effects. Some chemical agents like organophosphorus compounds (OPCs) including nerve agents and pesticides react with acetylcholinesteraseirreversibly. They inhibit normal biological enzyme action and result in accumulation of acetylcholineand show toxic effects andcholinergic symptoms. The process of Acetylcholinesterase (AChE) inhibition can be reversed by a nucleophilic agent to dephosphorylate and reactivate the enzyme. In this study, design and docking studies of 15 novel nitrone based onoximes as reactivators were performed by using AutoDock program. Then, more effective reactivatorsoximes in terms of binding energy and orientation within the active site were synthesized and evaluated *in-vitro* on human AChE (hAChE) inhibited by paraoxon and compared to standard hAChE reactivators (2-PAM and obidoxime). Our results used to design new derivatives of Oxim with better efficacy than 2-PAM and obidoxime. Syntheses of some selected bis-pyridiniumoximes based on the nitrones are underway.

## Introduction

Acetylcholinesterase (EC 3.1.1.7;AChE), one of the most important enzyme for human and other species, terminates cholinergic neurotransmission by splitting the neurotransmitter acetylcholine to choline and acetic acid (1, 2). The catalytic triad in this enzyme is composed of Ser203, His447 and Glu334 at the bottom of an about 20 Å active-site gorge, along with the lining of aromatic residues (3). The peripheral anionic site (PAS) at the entrance of the gorge comprising another set of aromatic residues, Trp286, Tyr72, Tyr124, and Tyr341 and the acidic Asp74, provides a binding site for allosteric modulators and inhibitors (4-6).

It is well known that organophosphorus compounds (OPCs) including nerve agents and pesticides react irreversibly with acetylcholinesterase by forming a covalent binding to the serine hydroxyl group within the active site (7, 8). Afterward, AChE is not able to fulfill its essential role and because of the accumulation of acetylcholine at cholinergic receptor sites, many medical disorders occur. This precipitates a cholinergic crisis characterized by saturation of muscarinic (*e.g.*, lacrimation, miosis, salivation) and nicotinic receptors (*e.g.*, muscle spasms), finally leading to death caused by respiratory failure (8-10).

**Figure 1:**
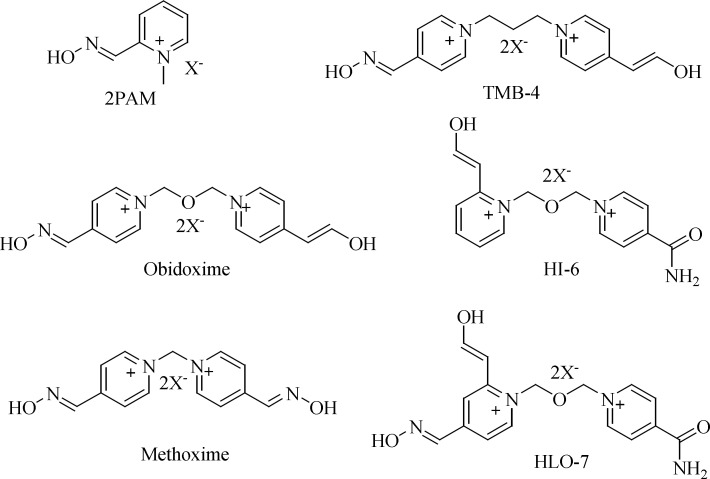
Commercially available AChE reactivators

The current standard treatment of poisoning with OPCs involves the administration of an anticonvulsant (*e.g.*, diazepam), a para sympatholytic agent (*e.g.*, atropine) and an oxime reactivator (*e.g.*, obidoxime, trimedoxime, methoxime, 2-PAM, HI-6, Hlo-7; (Figure 1) (11-13).

Due to their high nucleophilicity and secondary interactions of their cationic parts with the peripheral site of the enzyme, oximes can displace the phosphyl group from the catalytic serine and thus, restore the enzymeʹs vital function (14). Despite tremendous efforts to develop a universal reactivator, none is sufficiently effective against all known OP compounds (15-17). Additionally, the aged enzymes, a process that eliminates one of their substituents, are resistant to current oxime reactivators. A literature review indicates that there are a number of molecular docking studies that could be useful to further design of new potential reactivators (18-23). In continuation of our interest toward the progress of the green synthetic methodology of nitrone based oxims (24), new nitron based oximes involving pyridinium moiety as new drug candidate in reactivation of inhibited AChE was designed. In this study, 15 novel such oximes were designed and their docking studies were performed and the results were compared to those of obidoxime and 2-PAM as the current standard reactivatores. The synthesis of some selected novel oximes were then performed by two strategies followed by the *in-vitro* evaluation and the results were compared to those of obidoxime and 2-PAM.

## Experimental

*Molecular modeling*

Crystallographic coordinates of mAChE phosphorylated by tabun were taken from the Brookhaven Protein Data Bank (PDB code: 2Jf0) (25). All heteroatoms were removed from the PDB files and water molecules removed in the SPDB Viewer software (26). Based on our previous research, Docking calculations were carried out using Autodock (version 4) program package (27-36). Kollman partial charges were assigned to all protein atoms. Autogrid was carried out for the preparation of the grid map using grid boxes of 70-70-70 points of 0.375 Å spacing. A Lamarckian genetic algorithm (Amber force field) was used. A population of 150 individuals and 2,500,000 function evaluations were applied. Docking calculations were set to 30 runs. At the end of the calculation, the best superimposing poses were chosen for the analysis. The chemical structures of novel reactivators (Table 1) were built using Hyperchem software (Version 7, Hypercube Inc.).

*Chemistry*

3-Pyridine carboxaldehyde, hydroxylamine hydrochloride, Methyl iodide, Ethyl iodide, Butyl bromide and Benzyl bromide were purchased from Merk (Germany) and were used without further purification. The solvents were purchased from Merck (Germany) and dried and distilled before use. Diaminoglyoxim (DAG) was prepared according to the literature procedure (37). Obidoxime and 2-PAM were synthesized using reported methods (38, 39). Melting points were measured on an Electrothermal 9100 apparatus. Infrared spectra were recorded on Bruker Tensor 27 and Perkin Elmer spectrophotometers. ^1^H and ^13^C NMR spectra were recorded on Bruker 250 and 62.90 MHz instrument respectively. NMR spectra were obtained in DMSO-d_6_ using TMS as internal standard. Four different selected nitron based oximes were synthesized on the basis of preliminary computational predictions which are presented in (Table 2).

*In-vitro reactivation assay*

The reactivation ability was measured at 412 nm by using UV spectrophotometer (PERKIN-ELMER Lambda 5 and CECIL 8000) with a modified Ellman protocol. Recombinant hAChE (Sigma-Aldrich) was used throughout experiments. Pesticide paraoxon, diethyl-O-(4-nitrophenyl) phosphate, was purchased from (Sigma–Aldrich). Phosphate buffer (0.1 M, pH 7.4 at 37 °C), acetylthiocholine (1 mM) as substrate and DTNB (1 mM) as chromogen were used throughout all experiments. Paraoxon was used as organophosphorus inhibitor and the AChE reactivating capability of novel oximes, 2-PAM and obidoxime were examined against paraoxon-inhibited AChE. The enzyme was inhibited via the addition of excess of a paraoxon solution. To remove excess inhibitor, the reaction mixture was partitioned with six folded volume of hexane (40-42), and then the aqueous phase was separated by centrifugation. This was then followed by the addition of test oximes of appropriate concentration (10 µM or 100 µM) to start reactivation. The total volume of each assay mixture was 1.0 mL (phosphate buffer pH 7.4, 0.1 M). After 15 min of reactivation, the enzyme activity was assayed. All concentrations in the above assay mixture refer to the final concentrations. All assays were performed in triplicate and the reactivation data were expressed as average value ± standard deviation (SD). Percentage reactivation was calculated using the following equation (43).

%Reactivation = (E_r_ - E_i_/E_0_ – E_i_) × 100

Where E_0_ is the control enzyme activity at 0 min in absence of inhibitor and oxime, E_i_ is the inhibited enzyme activity in the absence of oxime, and E_r_ is the activity of reactivated AChE after incubation with the oxime test compounds.

*Synthesis of 4-amino-5-(hydroxyimino)-2-(1-pyridin-3-yl)-2, 5-dihydro-1H-imidazole 3-oxide (3)*

4-amino-5-(hydroxyimino)-2-(1-pyridin-3-yl)-2, 5-dihydro-1H-imidazole 3-oxide (3) was prepared by the reaction of DAG (1 mmol, 0.12 g), pyridine-3-carboxaldehyde (1.2 mmol, 0.11 ml) and p-toluene sulphonic acid (p-TSA) (0.2 mmol, 0.034 g) in ethanol. The reaction mixture was stirred at ambient temperature for 90 min after that white precipitates appeared in the reaction mixture. It was filtered and dried.

White powder; m.p 209-211 °C; yield 96%; IR (KBr, cm^-1^): 3327-3242 (NH_2_), 3159 (OH), 2851 (CH-Imidazole), 1715-1693 (C=N), 1605-1480 (Py-ring), 1330 (N=O), 1229 (C-N), 935-924 (N-O); ^1^HNMR (250 MHz, DMSO-d_6_); 5.63, 5.88 (s, H, NH_2_), 5.47, 5.55 (s, H, CH-Imidazole),7.42-8.71 (m, H-Ar, NH), 10.06, 10.21 (s, H, NOH) ppm. ^13^C NMR (62.5 MHz, DMSO-d_6_): 91.84, 124.24, 134.937, 135.59, 141.87, 148.64, 149.66, 150.93, 152.15 ppm.

*General procedure for the synthesis of compounds B*_1_ *-B*_2_ *and B*_5_

Compounds B_1_-B_2_ and B_5_ were synthesized by the N-alkylation reactions between pyridine-3-carboxaldehyde (5 mmol, 0.5 mL) and corresponding alkyl or benzyl halides (7 mmol) in dried acetone. Reaction mixture was heated at 60 °C for 6-8 h while vigorously stirred. After cooling, the separated precipitates were collected, washed with dry ether (20 mL) and dried. The powder 3-formyl-1-methylpyridinium iodide, dried in vacuo over phosphorus pentoxide. 3-formyl-1-butylpyridinium bromide product was not obtained under various conditions examined.

*3-Formyl-1-methylpyridinium Iodide (B*_1_ *)*

Yellow powder, m.p 173–176 °C, yield 85%; ^1^H NMR and ^13^C NMR spectra are consistent with the literature data (44).

*3-Formyl-1-ethylpyridinium Iodide (B*_2_ *)*

Yellow powder, m.p 132–135 °C, yield 76%, ^1^H NMR (250 MHz, DMSO-d_6_): 1.55-1.61 (t, CH_3_), 4.70-4.79 (q, CH_2_), 8.33-8.38 (dd, H-5), 8.98, 9.01 (d, H-4), 9.31, 9.33 (d, H-6), 9.56 (s, H-2), 10.17 (s, CH=O); ^13^C NMR (62.5 MHz, DMSO-d_6_): 16.61, 57.35, 128.98, 134.85, 145.06, 146.60, 148.46, 189.28.

*3-Formyl-1-benzylpyridinium Bromide (B*_5_ *)*

White powder, m.p 140–142 °C, 92%, ^1^H NMR (250 MHz, DMSO-d_6_): 6.00(s, CH_2_-benzyl), 7.44-7.63 (m, H-Ph), 8.35-8.38 (dd, H-5), 9.01, 9.04 (d, H-4), 9.43, 9.46 (d, H-6), 9.80 (s, H-2), 10.16 (s, CH=O); ^13^C NMR (62.5 MHz, DMSO-d_6_): 63.72, 129.30-129.93 (6C), 134.42, 135.24, 145.52, 146.76, 148.54, 189.10.

*General procedure for the synthesis of oximes 1B-2B and 4B-5B*

*First strategy*

A solution of **3** (1 mmol, 0.21), alkyl or benzyl halides (1.2 mmol) in DMF (3 mL) was stirred at room temperature for appropriate time. The progress of the reaction was monitored by TLC (ethyl acetate). Then, the mixture of the reaction was placed in a vacuum oven at 40 °C for 55 h to remove DMF. The precipitate was washed with dry acetonitrile and dry ethanol and then dried under vacuum.

**Figure 2.**
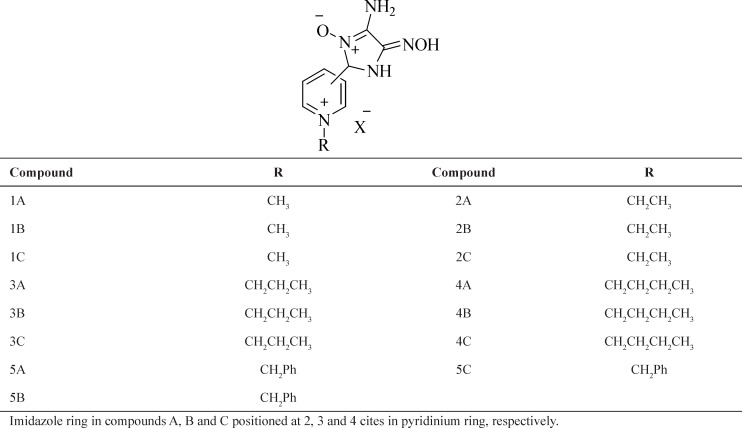
Docked structures of obidoxime (top) and 2PAM (Bottom) with the crystal structure of tabun-inhibited AChE (PDB code 2JF0). Hydrogen bonds and pi-pi interactions have been represented

**Figure 3:**
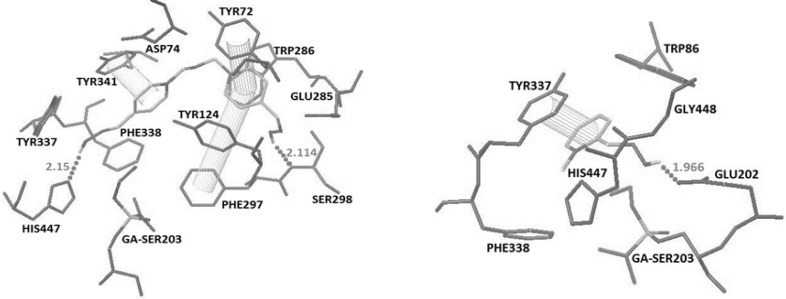
Docked structures of 4A (top) and 4B (Bottom) with Crystal structure of tabun-inhibited AChE (PDB code 2JF0). Hydrogen bonds and pi-pi interactions have been represented

**Figure 4:**
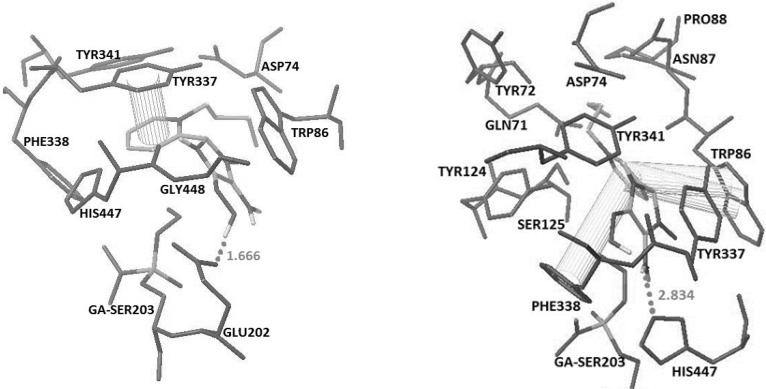
Docked structures of 4C (top) and 5B (Bottom) with Crystal structure of tabun-inhibited AChE (PDB code 2JF0). Hydrogen bonds and pi-pi interactions have been represented

**Scheme 1:**
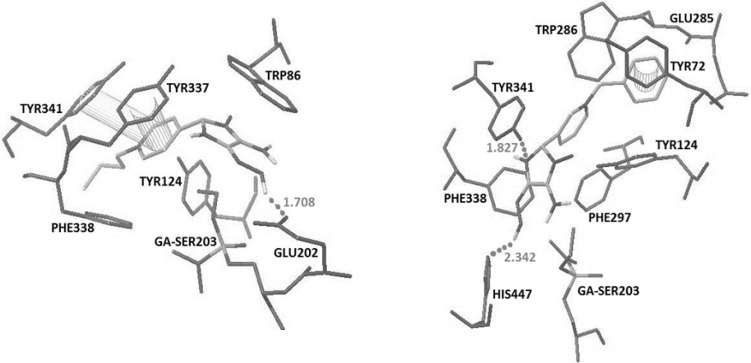
Synthetic scheme for the novel mono-pyridiniumoximes

**Table 1:** Structures of novel designed oximes

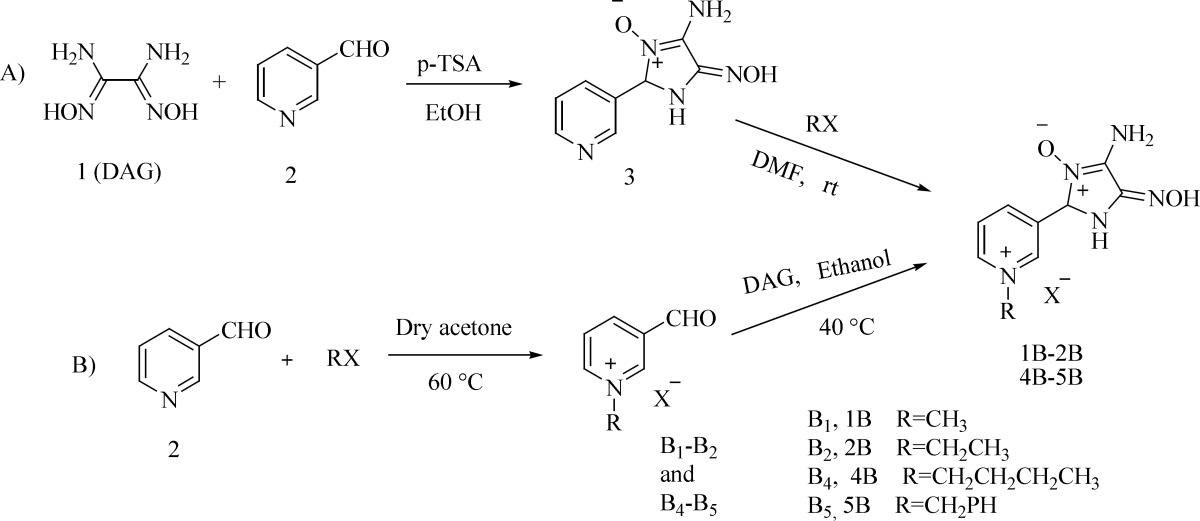

**Table 2:** Docking results of novel oximes based on nitrones by AutoDock 4 software

**Oximes**	**Intermolecular energy**	**vdw_hb_desolve_** **energy**	**Electrostatic energy**	**Total internal energy**	**Torsional energy**	**Unbound energy**	**Ki (µm)**	**Ref RMS**	**Building energy**
1B	-8.77	-8.61	-0.17	-0.67	1.19	-0.67	2.79	37.88	-7.58
2B	-9.62	-9.16	-0.16	-1.25	1.19	-1.25	0.67	39.1	-8.43
2C	-8.27	-8.16	-0.1	-1.34	1.19	-1.34	6.55	35.95	-7.07
3A	-9.41	-9.03	-0.38	-1.59	1.49	-1.59	1.58	37.17	-7.92
3B	-9.34	-9.28	-0.06	-1.23	1.49	-1.23	1.78	36.69	-7.53
3C	-8.73	-8.3	-0.44	-1.45	1.49	-1.45	4.92	36.36	-7.24
4A	-9.87	-9.47	-0.4	-1.98	1.79	-1.98	1.18	37.45	-8.08
4B	-9.66	-9.58	-0.07	-1.36	1.79	-1.36	1.71	36.28	-8.52
4C	-9.41	-8.98	-0.42	-1.53	1.79	-1.53	2.61	35.87	-7.62
5A	-10.94	-10.92	-0.02	-1.35	1.49	-1.35	0.12	37.21	-9.44
5B	-11.05	-11	-0.05	-1.47	1.49	-1.47	0.09	33.27	-9.56
Obidoxime	-10.98	-10.84	-0.14	-0.41	2.39	-0.41	0.51	36.34	-8.59
2-PAM	-6.2	-5.93	-0.27	-0.11	0.6	-0.11	0.07	37.37	-5.61

Due to the fact that compounds 1A, 1C, 2A, and 5C did not give favorable spatial orientations, calculation results of these oximes were deleted

**Table 3:** Reaction conditions for the synthesis of oximes 1-5B

**Oxime**	**R**	**X**	**Yield (%)** **First strategy (a)**	**Time (h)** **(a)**	**Yield (%)** **Second strategy (b)**	**Time (h)** **(b)**	**Mp (°C)**
1B	-CH_3_	I	64	2	33	40	158-160
2B	-CH_2_CH_3_	I	73	3	28	40	145-146
4B	-CH_2_(CH_2_)_2_CH_3_	Br	52	10	-	-	169-171
5B	-CH_2_Ph	Br	87	2	38	40	206-207

**Table 4:** Reaction conditions for the synthesis of compounds B_1_-B_2_ and B_4_-B_5_.

**Compound**	**R**	**X**	**Time (h)**	**Yield (%)**	**Mp (°C)**
B_1_	-CH_3_	I	7	85	173-176
B_2_	-CH_2_CH_3_	I	8	76	132-135
B_5_	-CH_2_Ph	Br	6	92	140-142
B_4_	-CH_2_(CH_2_)_2_CH_3_	Br	36	no reaction	-

**Table 5.** Reactivation ability of the tested oximes (reactivation (%) ± SD

**Reactivator**	**100 µm**	**10 µm**
2-PAM	11.2 ± 0.8	2.7 ± 0.5
obidoxime	60.3 ± 0.6	23.2 ± 0.3
1B	3.2 ± 0.8	0
2B	3.4 ± 0.3	0
4B	4.1 ± 0.4	1.4 ± 0.6
5B	7.8 ± 0.5	2.1 ± 0.3

Efficacy of the tested oximes in reactivation of paraoxon-inhibited AChE in comparison with 2-PAM and obidoxime. Time of reactivation by AChEreactivators 15 min; pH 7.4; temperature 37 °C. Values are average of triplicate runs with a standard deviation (SD).

*Second strategy*

Target oximes 1B-2B and 4B-5B were synthesized by the reaction between DAG (1 mmol, 0.12 g) and corresponding B_1_-B_2_ and B_5_ compounds (1.2 mmol) in dry ethanol under nitrogen atmosphere, which is sealed and heated at 40 °C with stirring for 40 h. The precipitates were collected, washed with dry ethanol and dried.

*Prepared oximes by the first strategy*

*4-amino-2-(1-methylpyridinium-3-yl)-5-(hydroxyimino)-2,5-dihydro-1H-imidazole 3-oxide Iodide (1B)*

Yellow powder; m.p 158-160 °C; yield 64%; IR (KBr, cm^-1^): 3307, 3240 (NH_2_), 3153 (OH), 2856 (CH), 1715, 1683 (C=N), 1642, 1474 (Py-ring), 1352, 1293 (N=O), 1221 (C-N), 958, 911 (N-O); ^1^H NMR (250 MHz, DMSO-d_6_): 4.40 (s, CH_3_), 5.63 (s, NH_2_), 6.72 (s, CH Imidazole),7.90-9.10 (m, pyridinium H, NH), 10.31 (s, NOH) ppm; ^13^C NMR (62.5 MHz, DMSO-d_6_): 48.04, 89.04, 127.59, 139.59, 141.67, 142.56, 143.44, 145.86, 151.31 ppm.

*4-amino-2-(1-ethylpyridinium-3-yl)-5-(hydroxyimino)-2,5-dihydro-1H-imidazole 3-oxide Iodide (2B)*

Yellowish powder; m.p 145-146 °C; yield 73%; IR (KBr, cm^-1^): 3412, 3254 (NH_2_), 3159 (OH), 2980 (CH), 1714, 1693 (C=N), 1652, 1480 (Py-ring), 1357 (N=O), 1230 (C-N), 954, 924 (N-O); ^1^H NMR (250 MHz, DMSO-d_6_): 1.51-1.56 (t, CH_3_), 4.63-4.71 (t, CH_2_), 5.72, 5.90 (s, NH_2_), 6.70, 6.84 (s, CH-Imidazole),7.70-9.21 (m, pyridinum H, NH), 10.14, 10.27 (s, NOH) ppm; ^13^C NMR (62.5 MHz, DMSO-d_6_): 16.42, 56.74, 89.45, 128.43, 140.57, 141.14, 142.96, 143.28, 145.24, 151.63 ppm.

*4-amino-2-(1-butylpyridinium-3-yl)-5-(hydroxyimino)-2,5-dihydro-1H-imidazole 3-oxide Bromide (4B)*

White powder; m.p 169-171 °C; yield 52%; IR (KBr, cm^-1^): 3335, 3241 (NH_2_), 3161 (OH), 2975 (CH), 1714, 1693 (C=N), 1606, 1434 (Py-ring), 1309 (N=O), 1230 (C-N), 935, 925 (N-O); ^1^H NMR (250 MHz, DMSO-d_6_): 0.83-0.96 (t, CH_3_), 1.23-1.39 (m, CH_2_), 1.93-1.98 

(m, CH_2_) 4.77-4.80 (t, CH_2_-N) 6.15 (s, NH_2_), 7.76 (s, CH-Imidazole), 8.32-9.97 (m, pyridinium H, NH), 10.54, (s, NOH) ppm; ^13^C NMR (62.5 MHz, DMSO-d_6_): 13.79, 19.18, 33.23, 79.40, 124.52, 129.25, 142.01, 144.01, 145.07, 148.19, 164.85, 171.20 ppm.

*4-amino-2-(1-benzylpyridinium-3-yl)-5-(hydroxyimino)-2,5-dihydro-1H-imidazole 3-oxide Bromide (5B)*

White Powder; m.p 206-207 °C; yield 87%; IR (KBr, cm^-1^): 3392, 3290 (NH_2_), 3155 (OH), 3001 (CH), 1644 (C=N), 1578, 1473 (Py-ring), 1341 (N=O), 1255 (C-N), 951, 936 (N-O); ^1^H NMR (250 MHz, DMSO-d_6_): 6.03(s, CH_2_-benzyl), 6.15 (s, NH_2_), 7.44-7.62 (m, Ph, CH and NH-Imidazole), 8.37-8.42 ( dd, H-5), 9.20, 9.23 (d, H-4), 9.41, 9.43 (d, H-6), 10.11 (s, H-2), 10.54 (s, NOH) ppm; ^13^C NMR (62.5 MHz, DMSO-d_6_): 63.86, 124.54, 129.18-130.10 (6C), 133.93, 141.67, 144.23, 144.66, 147.74, 164.41, 170.77 ppm.

## Results and Discussion

*Docking studies*

The novel oximes (Table 1) together with the standard oximes pralidoxime (2-PAM) and obidoxime as references were docked inside active site of the crystal structure of tabun-inhibited AChE and all their reasonable binding orientations for the mAChE reactivation were investigated. Then, the best superimposing poses were chosen for the analysis. The docking results of novel nitroneoximes compared to two current oximes, obidoxime and 2-PAM, are presented in Table 2.

The recorded binding energy for obidoxime and 2-PAM were -8.59 and -5.61 respectively. Obidoxime interacted with His447 and Ser298 by making hydrogen bonds and additional pi-pi interactions with Tyr341, Tyr72 and Phe297 while 2-PAM interacted with Glu202 by making a hydrogen bonds and an additional pi-pi interactions with Tyr337 (Figure 2).

The docking results showed that in oximes 3A and 4A where the imidazole ring was positioned at position 2 on the pyridinium ring, hydrogen bonding interaction with Glu202 and a pi-pi interaction with Tyr337 are the major interaction forces (Figure 3).

In comparison to oximes 3A and 4A, 5A formed a hydrogen bonding interaction with His447 and displayed three pi-pi interactions with aromatic residues Trp86, Phe338 and Tyr72. The high binding energy of 5A is caused by the presence of additional pi-pi interactions. Nevertheless, oximes 1A and 2A didn’t have favorable spatial orientation.

By changing the position of imidazole group on the pyridinium ring from 2 to 3 or 4, the interaction patterns are changed.

Oximes 1-4B involving the imidazole group at position 3, show a hydrogen bonding interaction with His447 and two pi-pi sandwiches with Trp86 and Phe338, while 5B oxime indicates interaction with His447 and Tyr341 by making two hydrogen bonds and pi-pi interaction with PAS residue of Tyr72 (Figures 3 and 4).

In oximes 2-4 C where the imidazole group is attached to the position 4 on the pyridinium ring, a hydrogen bonding interaction with Glu202 and two pi-pi interactions with aromatic residues Tyr337 and Tyr341 have been noticed (Figure 4) whereas oximes 1C and 5C have no favorable spatial orientation. It is of interest to note that as a general trend, in most cases for all of the oximes A, B and C, the more chain length of the alkyl substitute on the pyridiniumnitrogrn, the higher the binding energy.

As is clear from the docking results, all of the new nitroneoximes present better binding energies than 2-PAM and are comparable with obidoxime except those including 1A, 2A, 1C and 5C that have unfavorable position and orientation. Among the evaluated promising oximes, the binding energies of oximes 1-5 B are better than those of 1-5 A and 1-5 C, inside the active site of the gorge.

*Synthesis of novel oximes as reactivators*

According to the docking simulation, oximes 1-5 B were the best in terms of binding energy and orientation within the active site. To test the docking results, the synthesis and investigation of the reactivation ability of selected oximes, except 3B that had relatively less binding energy, was designed. Therefore, two strategies designed to achieve the novel nitroneoximes as shown in Scheme 1. DAG, was easily synthesized from the reaction of glyoxal and hydroxylammonium chloride according to reference procedure. In the first strategy, 4-amino5(hydroxyimino)-2-(1-pyridin-3-yl)-2, 5-dihydro-1H-imidazole 3-oxide (3) was prepared by the reaction of DAG and pyridine-3-carboxaldehyde in the presence of p-toluene sulphonic acid (p-TSA) in ethanol to obtain nitrone 3 in 96% yield. The structure of this product was assigned by IR, ^1^H NMR and ^13^C NMR. The strong absorption band at 2851 cm^-1^ in the IR spectrum, characteristic peak in 91.84 ppm in the ^13^C spectrum and characteristic signals at 5.47, 5.55 ppm in the ^1^H NMR spectrum should be assigned to the spiro carbon in imidazole ring. Also, the other peaks in ^1^H NMR and ^13^C NMR confirm that desired product was formed (24).

Afterward, the N-alkylation reactions of compound 3 with alkyl or benzyl halides were carried out under the mild condition, at room temperature, in DMF solvent for 2–12 h (Table 3). The structural elucidations of the products (1B-2B and 4B-5B) were assigned by IR, ^1^H NMR and ^13^C NMR. The ^1^H NMR spectrum of 5B consisted of three singlets for the benzyl methylene, NH_2_ and NOH at 6.03, 6.15 and 10.54 ppm, respectively. The NH_2_ protons peak was exchangeable with D_2_O. Characteristic signals at 7.44-7.62 ppm corresponding to the phenyl protons, CH and NH of imidazole ring that NH peak was exchangeable with D_2_O, and 8.37-10.11 ppm, assignable to the pyridinium protons. The ^13^C NMR and DEPT 135 spectra of 5B showed characteristic resonances at 63.8 (CH_2_-Ph), 124.54 (spiro carbon), 129.18-130.10 (6C, Ph), 133.93-147.74 (5C, pyridinium) along with 164.41 and 170.77 that are assigned to the two alkene carbons at imidazole ring. The ^1^H and ^13^C NMR spectra of 1B-2B and 4B were more or less similar to those of 5B except for the alkyl substituents, which exhibited characteristic signals with appropriate chemical shifts. Also, it should be pointed out that compound 3 and butyl bromide reacted more smoothly than others, which need longer reaction time and gave lower yield of product.

In the second strategy, oximes B_1_-B_2_ and B_5_ were synthesized by the N-alkylation reactions between pyridine-3-carboxaldehyde and corresponding alkyl or benzyl halides in dried acetone (Table 4). The structure of the isolated product B_5_ was deduced on the basis of ^1^H and ^13^C NMR spectroscopy. The ^1^H NMR spectrum of this compound exhibited a sharp singlet at 6.00 ppm due to the benzyl methylene, a singlet for CHO at 10.16 ppm along with characteristic resonances for the phenyl ring at 7.44-7.63 ppm and pyridinium ring at 8.35-9.80 ppm. The ^13^C NMR spectrum of B_5_ showed 13 distinct resonances at 63.72 (CH_2_-Ph), 129.30-129.93 (6C, phenyl), 134.42-148.54 (5C, pyridinium), 189.10 ppm (CHO) in agreement with the proposed structure. The ^1^H and ^13^C NMR spectra of B_2_ were similar to those of B_5_ except for the ethyl substituent, which exhibited characteristic signals with appropriate chemical shifts. Also, ^1^H NMR and ^13^C NMR results of B_1_ are consistent with literature data (37). However, the preparation of 3-formyl-1-butylpyridinium bromide was not successful although the reaction was repeatedly performed under various conditions. Then, target oximes 1B-2B and 5B were synthesized by the reactions between DAG and corresponding B_1_-B_2_ and B_5_oximes in dry ethanol under nitrogen atmosphere. The ^1^H and ^13^C NMR data of the resulting oximes were similar to those obtained by the first strategy. Also, the second strategy afforded lower yields than the first strategy, even when the reaction times were increased to 40 h.

*In-vitro studies*

In order to assess the reactivation abilities of the oximes, paraoxon was used to inactivate AChE. The reactivation of phosphorylated AChE by oximes is strongly dependent upon the chemical structure of the reactivators, OP oximes and source of the enzyme AChE (45). Human recombinant acetylcholinesterase was used as an enzyme source in the present study. As shown in Table 5, the potencies of the prepared oximes were compared with the two commercials used AChEreactivators, 2-PAM and obidoxime.

Concerning paraoxon-inhibited AChE, commercial compounds 2PAM and obidoxime presented the expected results (46, 47). From these data it is evident that four newly synthesized oximes (1B-2B and 4B-5B) are less active than obidoxime and also 2-PAM, in reactivating paraoxon-inhibited AChE.

Regarding paraoxon-inhibited hAChE, 2-PAM (2.7%), 4B (1.4%), 5B (2.1%) and 1B, 2B (0%) were all inefficient reactivators at 10 µM. However, obidoxime was found to be potent paraoxon-reactivator (23.2%). While, all of our novel reactivators showed some reactivation ability at a concentration of 100 µM, their ability 1B (3.2%), 2B (3.4%), 4B (4.1%) and 5B (7.8%) were obviously less than obidoxime (60.3%) and 2-PAM (11.2%). Moreover, among the novel synthesized oximes, compound 5B was found to be the best but did not reach the reactivator ability of 2-PAM oxime.

Finally, it might be of interest to note that although none of these novel oximes were able to surpass the reactivation efficacy of obidoxime and 2-PAM for the reactivation of hAChE inhibited by paraoxon at concentrations of 10 µM and 100 µM, bis-pyridinium derivatives of these newly introduced oximes might be promising. The preliminary docking studies on such bispyridinium species indicates that it is logical to practically evaluate this proposal.

## Conclusion

In summary, docking studies on some novel nitrone oximes and two current standard reactivatores, obidoxime and 2-PAM, were performed. The screening results showed that all of the new oximes possess better binding energies than 2-PAM and were comparable with obidoxime. Moreover, the binding energies of oximes 1-5 B were better than those of corresponding 1-5 A and 1-5 C in terms of binding energy and orientation within the active site.

Superior oximes, then, were synthesized and evaluated for their reactivation efficacy against paraoxon-inhibited AChE. None of the tested oximes have shown better reactivation efficiencies than those of the standard oximes. It is suggested that bis-pyridinium derivatives of these oximes family may have higher potency than the reported cases here. The synthesis and evaluation of bis-pyridiniumoximes based on the nitrones are underway.
